# *KRAS* ^G12C^ mutation‐induced TOPK overexpression contributes to tumour progression in non‐small cell lung cancer

**DOI:** 10.1111/jcmm.17640

**Published:** 2023-05-24

**Authors:** Chang Cai, Shuo Yao, Yanmei Zou, Hui Lu, Xiuqiong Chen, Yali Wang, Kun Zheng, Feng Zhu, Yihua Wang, Hua Xiong, Junfei Zhu

**Affiliations:** ^1^ Department of Respiratory and Critical Care Medicine The First Affiliated Hospital of Wenzhou Medical University Wenzhou China; ^2^ Department of Oncology, Tongji Hospital, Tongji Medical College Huazhong University of Science and Technology Wuhan China; ^3^ Wuhan Children's Hospital (Wuhan Maternal and Child Healthcare Hospital), Tongji Medical College Huazhong University of Science and Technology Wuhan China; ^4^ Cancer Research Institute The Affiliated Hospital of Guilin Medical University Guilin China; ^5^ Biological Sciences, Faculty of Environmental and Life Sciences University of Southampton Southampton UK; ^6^ Institute for Life Sciences University of Southampton Southampton UK; ^7^ NIHR Southampton Biomedical Research Centre University Hospital Southampton Southampton UK; ^8^ Department of Respiratory Medicine Taizhou Central Hospital (Taizhou University Hospital) Taizhou China

**Keywords:** *KRAS* ^G12C^, NSCLC, proliferation, TOPK

## Abstract

*KRAS* mutation is the most frequent type of genetic mutation in non‐small cell lung cancer (NSCLC), especially in lung adenocarcinoma. However, *KRAS* mutation can affect many biological processes and the mechanisms underlying *KRAS* mutation‐mediate carcinogenesis in NSCLC have not been fully understood. In this research, we found that *KRAS* ^G12C^ mutation was associated with the upregulation of T‐LAK cell‐originated protein kinase (TOPK), which is a well‐known serine/threonine MAPK‐like protein kinase implicated in tumorigenesis. The overexpression of TOPK significantly promoted the malignant phenotype of A549 cells, and TOPK silencing impaired the malignant phenotype with *KRAS* ^G12C^ mutation. Moreover, we demonstrated that TOPK level was regulated by MAPK/ERK signalling and the transcription factor Elk1. TOPK was also found to promote the activation of NF‐κB signalling in A549 cells with *KRAS* ^G12C^ mutation via facilitating the phosphorylation of TAK1. In the in vivo tumorigenesis model, the administration of TOPK inhibitor OTS514 enhanced the anticancer effect of 5‐FU, and the combinatory use of OTS514 and *KRAS* ^G12C^ inhibitor AMG510 showed synergistic anti‐tumour effect. These results suggest that KRAS‐TOPK axis contributes to the progression of NSCLC and targeting this axis could synergize with anticancer effect of the existing chemotherapeutics.

## 1 INTRODUCTION

As one of the most frequent type of malignant tumours, lung cancer poses a serious health threat to public health worldwide.^1^ According to histopathological classification, lung cancers are classified into small cell lung cancer (SCLC) and non‐small cell lung cancer (NSCLC), and NSCLC accounts for about 85% of the total diagnosed cases of lung cancer.^2^ For patients in early stage, surgical resection is the optimal approach for the treatment, and chemotherapy remains as the mainstay treatment for advanced NSCLC.^3^ However, the 5‐year survival rate of NSCLC is only 17.5%.^4^ Therefore, exploring the pathogenesis and molecular mechanisms underlying the progression of NSCLC is crucial to develop targeted therapies for more effective treatment of NSCLC.

The dysregulated cell proliferation in cancer cells is mainly caused by the accumulation of genetic mutations. Kirsten rat sarcoma viral oncogene homologue (*KRAS*) mutation is one of the most prevalent genetic mutations in NSCLC, and its activation has been described in approximately 35% cases of NSCLC.^5^ Wild type KRas protein cycles between two different states: GDP‐bound inactive state (KRas‐GDP) and GTP‐bound active state (KRas‐GTP). The prevalent mutations including G12, G13, and Q61 lock the mutated KRas protein in a constitutively active GTP‐bound state, leading to a persistent oncogenic signalling.^6^ Glycine‐to‐cysteine (G12C) mutation is a dominant *KRAS* mutation accounting for nearly 44% of *KRAS* mutations in NSCLC patients, which has been found to be extremely common in NSCLC patients with smoking history.^7^ Recently, AMG510 has been developed as the first *KRAS* ^G12C^ inhibitor, and it shows strong anti‐cancer efficiency and suppresses the progression of NSCLC.^8^ These findings suggest that researches that targeting *KRAS* ^G12C^ mutation can serve as a promising strategy in the treatment of NSCLC.

Recently, T‐LAK cell‐originated protein kinase (TOPK), also known as PDZ‐binding‐kinase (PBK), has been reported as a critical oncogenic protein in multiple cancers.^9^, ^10^, ^11^, ^12^ TOPK has been reported to be upregulated in a variety of cancers, such as oral cancer, breast cancer, leukaemia and prostate cancer,^9^, ^10^, ^11^, ^12^ whereas its expression is relatively low in normal tissues. TOPK is implicated in various physiological and biological processes, such as autophagy, apoptosis, and cell proliferation.^13^, ^14^, ^15^ In lung cancer, the upregulation of TOPK was reported to promote chemical‐resistance towards EGFR tyrosine kinase inhibitors,^16^ and facilitate hypoxia‐induced epithelial‐mesenchymal transition and the invasion of NSCLC cells.^17^ However, the molecular mechanism underlying TOPK upregulation in NSCLC remained to be elucidated.

In the current study, we explored the expression of TOPK in lung tissues of NSCLC patients with *KRAS* ^G12C^ mutation. We found that TOPK expression was significantly higher in tissues with *KRAS* ^G12C^ mutation compared with those with wild type *KRAS* gene. We further showed that TOPK was induced by the excessive activation of transcription factor Elk1 through MAPK/ERK signalling. Furthermore, TOPK facilitated the activation of NF‐κB signalling via promoting the phosphorylation of TAK1 in NSCLC cells. The administration of TOPK inhibitor OTS514 enhanced the anticancer effect of 5‐FU in the in vivo tumorigenesis model, and the combinatory use of OTS514 and *KRAS* ^G12C^ inhibitor AMG510 showed synergistic anti‐tumour effect. In summary, these results suggest that the constitutive activation of KRAS‐TOPK axis contributes to the malignant progression of NSCLC, and targeting this axis could serve as a targeted therapy for NSCLC treatment.

## 2 METHODS

### 2.1 RNA‐seq analysis and bioinformatics analysis

The RNA samples from NSCLC cells with *KRAS* ^G12C^ mutation and *KRAS* ^WT^, were examined by RNA‐seq analysis using the service from MDL Biotech. Prognosis and survival data was obtained from The Cancer Genome Atlas website (TCGA) database (https://cancergenome.nih.gov/).

For RNA‐seq, 3 replicates of each condition were subjected to total RNA total RNA extraction using Trizol reagent. Samples containing 300 ng/μl total RNA were used for library preparation using the TruSeq Stranded mRNA Library Prep Kit (Illumina), according to the manufacturer's protocol. Deep sequencing was performed using HiSeq 2000 Sequencing System (Illumina). For each sample, at least 20 million reads were generated. Raw read alignment was performed by tophat2 v2.1.0 using the Mus musculus GRCm38.p4 genome version (with the parameters “–no‐novel‐junctions”, “no‐coverage‐search option”, “library‐type = fr‐firststrand” and “–G” when specifying the genome file). The overall alignment rates were ~99%. Cufflinks v2.2.1 (with the –G parameter and the same GTF from tophat analysis) was used to derive expression (FPKM) values from the alignments using the same genome/annotation. Cuffmerge package (with the ‐g and ‐s parameters, the same GTF and the reference FASTA) was then used to merge the resulting transcripts before performing differential gene expression by Cuffdiff package (using the ‐b parameter with reference FASTA, and with the ‐‐max‐bundle‐frags 5,000,000 and ‐u parameters). FDR‐adjusted *p* value after Benjamini‐Hochberg correction for multiple‐testing were used as the statistics to define the differential expression. Genes with FDR‐adjusted *p* < 0.05 are considered to be differentially expressed.

### 2.2 Cell culture and stable cell line generation by lentivirus

Human NSCLC cell line A549 was purchased from Type Culture Collection of Chinese Academy of Sciences. Cells were cultured in RPMI‐1640 containing 10% fetal bovine serum (FBS; Gibco), 100 U/ml of penicillin and 100 μg/ml of streptomycin in a humidified incubator containing 5% CO_2_ at 37°C. The A549 cell lines with stable expression of *KRAS* ^WT^ or *KRAS* ^G12C^ were generated as previous described.^18^ The silencing and overexpression lentiviruses of TOPK were synthesized by Genscript Biotech company. The sequences used for TOPK silencing are as follow: (Mut‐KD1: ATT AGT GCA TAC AGA GAA GAG TT), Mut‐KD2: GTC TGT GTC TTG CTA TGG AAT. To generate stable cells with TOPK silencing or overexpression, cells were infected with the corresponding recombinant lentivirus at a MOI (multiplicity of infection) = 5 in the presence of 10 μg/ml polybrene (Sigma, tr‐1003‐g). Infected cells were selected with 1.0 μg/ml puromycin for 2 weeks to eliminate the uninfected cells before further experiment.

### 2.3 Clinical samples, xenograft model and Immunohistochemistry (IHC)

Ten *KRAS* ^WT^ NSCLC or ten *KRAS* ^G12C^ NSCLC tissues and individual normal paratumor tissues were collected from our hospital (Tongji Hospital, Tongji Medical College, Huazhong University of Science and Technology). The samples were frozen in −80 degree deep freezer. The study was approved by the Medical Ethics Committee, Tongji Hospital, Tongji Medical College, Huazhong University of Science and Technology.

To establish the xenograft model, 4 weeks old female nude mice were obtained from Vital River Laboratories. TOPK overexpression *KRAS* ^WT^ A549 cells or TOPK silencing *KRAS* ^G12C^ A549 cells were injected subcutaneously into the nude mice (5 × 106 per mice, 6 mice per group). The tumour volume was continuously monitored for 7 weeks, and the mice were sacrificed on week 7 for tumour weight measurement. For drug experiments, *KRAS* ^G12C^ A549 cells were injected subcutaneously into the nude mice. 2 weeks later the mice received intraperitoneal injection of drug or drug combinations two times a week for another 4 weeks. Mice were then sacrificed, the tumour volume and tumour weight were analysed. The xenograft tumour samples and clinical samples were collected for IHC analysis of Ki‐67 analysis as described previously,^12^ and anti‐Ki67 antibody (1: 500, #9449, CST) was used for the IHC staining. The animal experiments were approved by the Institutional Animal Care and Use Committee, Tongji Hospital, Tongji Medical College, Huazhong University of Science and Technology.

### 2.4 Quantitative real time‐PCR (qRT‐PCR) and luciferase reporter assay

Quantitative real time‐PCR was performed as previously reported.^19^ TRIzol reagent (Invitrogen) was used for total RNA extraction. Reverse transcription was performed using PrimeScript RT Reagent Kit (TaKaRa). The resulted cDNA was quantified using SYBR Green Master mix (TaKaRa) on a 7500 Real Time PCR System (Applied Biosystems). All the kits were used in the accordance of the manufacturer's protocol. The primers were used as follow: TOPK‐Forward: 5′‐GCG CGA CTT TTT GAA AGC CA‐3′ and TOPK‐Reverse: 5′‐ACG GAG AGG CCG GGA TAT TT‐3′; GAPDH‐Forward: 5′‐ATG GAA ATC CCA TCA CCA TCTT‐3′ and GAPDH‐Reverse: 5′‐CGC CCC ACT TGA TTT TGG‐3′.

### 2.5 Dual luciferase reporter assay

The promoter activities of TOPK and NF‐κB were examined by dual luciferase reporter assay. The luciferase reporters containing corresponding prompters were provided by Sangon Biotechnology Co., Ltd. The reporter plasmid and Renilla luciferase (hRlucneo) control plasmid were co‐transfected into *KRAS* ^G12C^ A549 cells in a 12‐well plate (1 × 105 cells/well) using Lipofectamine 3000 reagent according to the manufacturer's instructions (Invitrogen, L3000001). 48 h post transfection, the relative luciferase activities were measured using Dual‐Luciferase Reporter Assay Kit (Promega, E1910) on a luminescence microplate reader (Infinite 200 PRO; Tecan).^20^

### 2.6 CCK8 proliferation assay

Cell Counting Kit 8 (Dojindo Laboratory) was used for cell proliferation analysis according to the manufacturer's protocol. Cells were seeded in to a 96‐well plate at a density of 1500 cell/well and cultured in a humidified cell culture incubator for 0, 24, 48, 72 and 96 h, respectively. 10 μl CCK8 reaction solution was added to the cell culture at indicated time point and incubated for 1 h in a humidified cell culture incubator. The light absorption value (OD value) in each condition was captured at 450 nm wavelength on a Synergy H1 microplate reader.

### 2.7 Colony formation assay

Colony formation assay was performed as described.^21^ Briefly, TOPK overexpressed *KRAS* ^WT^ cells or TOPK silenced *KRAS* ^G12C^ cells were seeded in 6‐well plate (2000 cells/well) with indicated drug treatment. The culture medium was changed every 3 days. After 10 days, cells were fixed with 4% paraformaldehyde and stained with 0.5% crystal violet (Beyotime) for 20 min. The number of colonies was counted and the morphology of the colonies was photographed under Leica AM6000 microscope (Leica).

### 2.8 Apoptosis analysis

For apoptosis analysis, Annexin V‐fluorescein isothiocyanate Apoptosis Detection Kit (BD Biosciences) was used according to the manufacturer's instruction, followed by the analysis by FACSan flow cytometry (BD Biosciences). For TUNEL staining, TUNEL Assay Kit (HRP‐DAB, Elasbscince) was used to stain the apoptotic cells, and the images were captured under Leica AM6000 microscope.

### 2.9 Transwell and migration invasion assay

Cells with different treatments were trypsinized and re‐suspended in serum‐free medium. The transwell upper chamber coated with Matrigel (BD Biosciences) was used for invasion assay. The transwell chamber without Matrigel coating was used for migration assay. 2.5 × 105 cells were inoculated into the upper chamber in serum‐free medium and 500 μl of 10% serum‐containing medium was added to the lower chamber. After 24 h, culture medium was discarded and the cells were fixed with 4% paraformaldehyde at room temperature for 10 min and stained with 0.5% crystal violet (Beyotime) for 20 min. Cells were photographed under Leica AM6000 microscope.

### 2.10 Western blotting analysis and immunoprecipitation (IP)

RIPA buffer (Sigma) was used to extract the total protein. Concentration of protein was measured by BCA protein quantification kit (Thermo Scientific). Western blot and IP were performed as described.^21^ The antibodies used in the research were as follow: anti‐p65 (1:1000, #8242, CST), anti‐phos‐p65 (1:1000, #3033, CST), anti‐IKKβ (1:1000, #8943, CST), anti‐phos‐IKKβ (1:1000, #2694, CST), anti‐IκBα (1:800, #4814, CST), anti‐phos‐IκBα (1:800, #2859, CST), anti‐TOPK (1:500, #sc390399, Santa Cruz), anti‐MEKK1 (1:500, PA5‐43209, Invitrogen), anti‐phos‐MEKK1 (1:500, PA5‐12755, Invitrogen), anti‐MKK3 (1:1000, #8535, CST), anti‐phos‐MKK3 (1:1000, #12280, CST), anti‐p38 (1:1000, #8690, CST), anti‐phos‐p38 (1:1000, #4511, CST), anti‐PI3K (1:1000, #4255, CST), anti‐phos‐PI3K (1:1000, #17366, CST), anti‐Akt (1:1000, #9272, CST), anti‐phos‐Akt (1:1000, #9611, CST), anti‐Raf (1:1000, #9422, CST), anti‐phos‐Raf (1:1000, #9427, CST), anti‐MEK1 (1:1000, #2352, CST), anti‐phos‐MEK1 (1:1000, #9127, CST), anti‐ERK (1:1000, #4695, CST), anti‐phos‐ERK (1:1000, #4370, CST), anti‐Elk1 (1:500, #sc25986, Santa Cruz), anti‐phos‐Elk1 (1:500, #sc8406, Santa Cruz), anti‐TAK1 (1:1000, #5206, CST), anti‐phos‐TAK1 (1:1000, #9339, CST), anti‐KRAS (1:1500, ab275876, Abcam), and anti‐GAPDH (1:1000, #sc47724, Santa Cruz).

### 2.11 Statistical analyses

The data in the study were expressed as means ± SD from at least three independent experiments. Survival of NSCLC patients was described by Kaplan–Meier and analysed by log‐rank test. The statistical difference between two groups was compared using unpaired student's *t* tests. Comparisons among multiple groups were analysed using one‐way analysis of variance (ANOVA) with Tukey's post hoc test for pairwise comparison. Comparisons of data at multiple time points were examined using two‐way ANOVA. *p* < 0.05 was considered to be statistically significant.

## 3 RESULTS

### 3.1 TOPK is upregulated in NSCLC tissues and cells with *KRAS* ^G12C^ mutation

**FIGURE 1:**
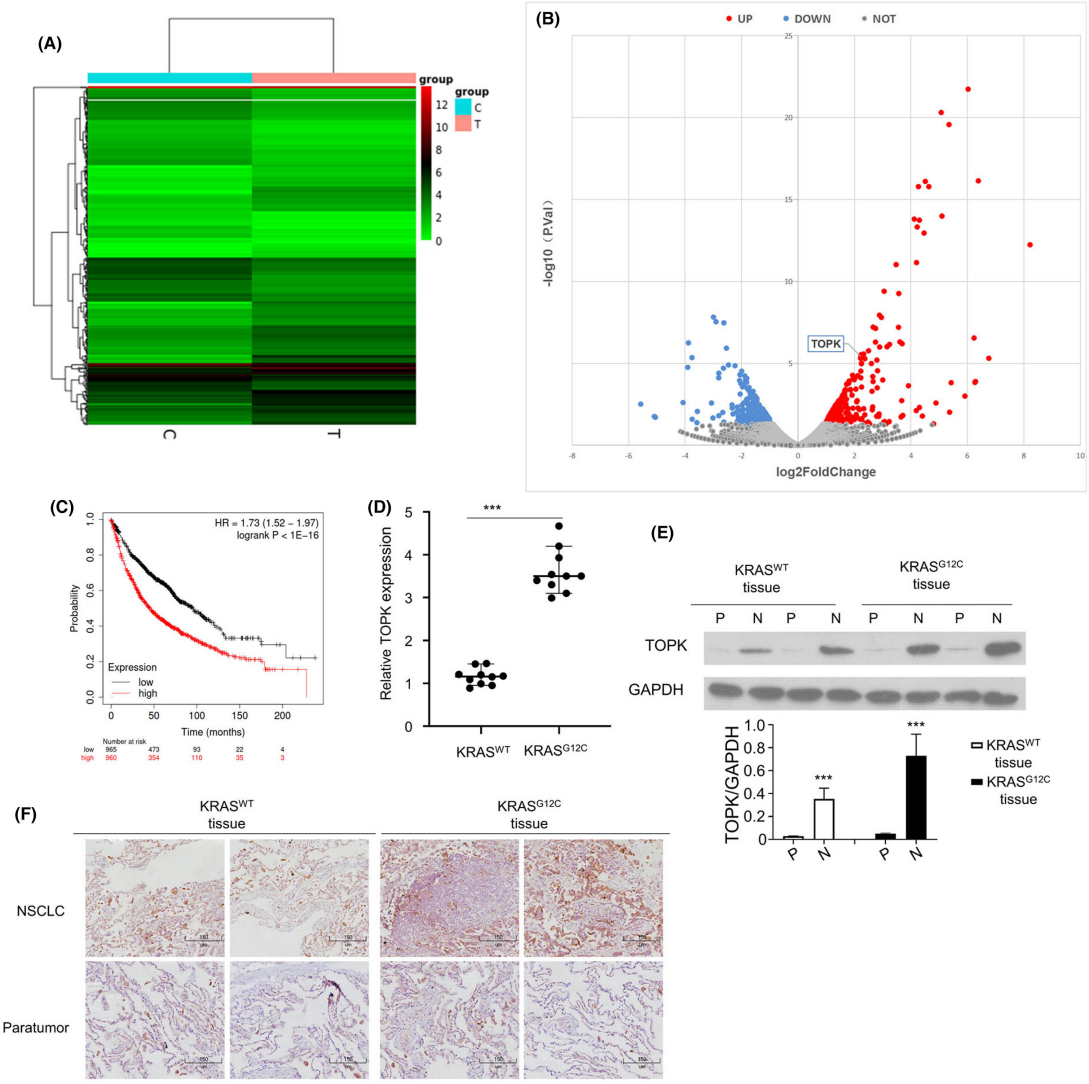
TOPK is upregulated in NSCLC tissues and cells with *KRAS* ^*G12C*^ mutation. (A) Heat map of differentially expressed transcripts in *KRAS* ^WT^ (C) or *KRAS* ^G12C^ (T) A549 NSCLC cells. (B). Volcano plot of differentially expressed transcripts in *KRAS* ^WT^ or *KRAS* ^G12C^ A549 cells. (C). Kaplan–Meier curves for the overall survival of NSCLC patients with low vs. high level of TOPK. (D). qPCR analysis of TOPK expression in NSCLC tissues from patients with *KRAS* ^WT^ or *KRAS* ^G12C^ mutation. (E). Protein levels of TOPK in para‐cancerous (P) or NSCLC (N) tissues of *KRAS* ^WT^ or *KRAS* ^G12C^ patients were determined by Western blot. (F). TOPK levels were examined by IHC in xenograft tissues

To explore the differentially expressed genes between A549 cells with *KRAS* ^G12C^ mutation and *KRAS* ^WT^, we established A549 cells stably expressing *KRAS* ^WT^ or *KRAS* ^G12C^. The stable expression of *KRAS* ^WT^ or *KRAS* ^G12C^ was examined by Western blot (Figure S1A). Cells were subjected to RNA‐seq analysis, and differentially gene expression analysis (fold change of at least 2.0 and adjusted *p* value of <0.05) showed that TOPK was among the genes upregulated in *KRAS* ^G12C^ A549 cells (Figure 1A,B, Table S1). To analyse the association of TOPK with the survival of NSCLC patients, we extracted the survival data of a cohort of NSCLC patients from The Cancer Genome Atlas website (TCGA) database. As shown in Figure 1C, Kaplan–Meier survival analysis suggested that a high level of TOPK was associated with a poorer 5‐year overall survival (OS) in NSCLC patients, which was consistent with the previous reports about the impaired survival in NSCLC patients with *KRAS* ^G12C^ mutation.^22^, ^23^ To confirm the finding, we collected 10 *KRAS* ^WT^ NSCLC and 10 *KRAS* ^G12C^ NSCLC tissues and demonstrated the overexpression of TOPK in *KRAS* ^G12C^ tissues compared with *KRAS* ^WT^ tissues by RT‐qPCR (Figure 1D). We also compared the protein levels of TOPK between normal lung tissues and the NSCLC tissues with *KRAS* ^WT^ or *KRAS* ^G12C^. Western blot and IHC staining results showed that TOPK was upregulated in all NSCLC tissues when compared to the normal lung tissues, and there was a relatively higher expression in NSCLC tissues *KRAS* ^G12C^ mutation when compared to the NSCLC tissues with *KRAS* ^WT^ tissues (Figure 1E,F). Consistently, in the A549 cells with *KRAS* ^G12C^ mutation, the TOPK protein level was significantly higher when compared to that of wild type cells (Figure S1B). Taken together, these findings suggest that the upregulation of TOPK expression is positively correlated with *KRAS* ^G12C^ mutation in NSCLC.

### 3.2 TOPK is indispensable for the malignant phenotype of NSCLC cells with *KRAS* ^G12C^ mutation

**FIGURE 2:**
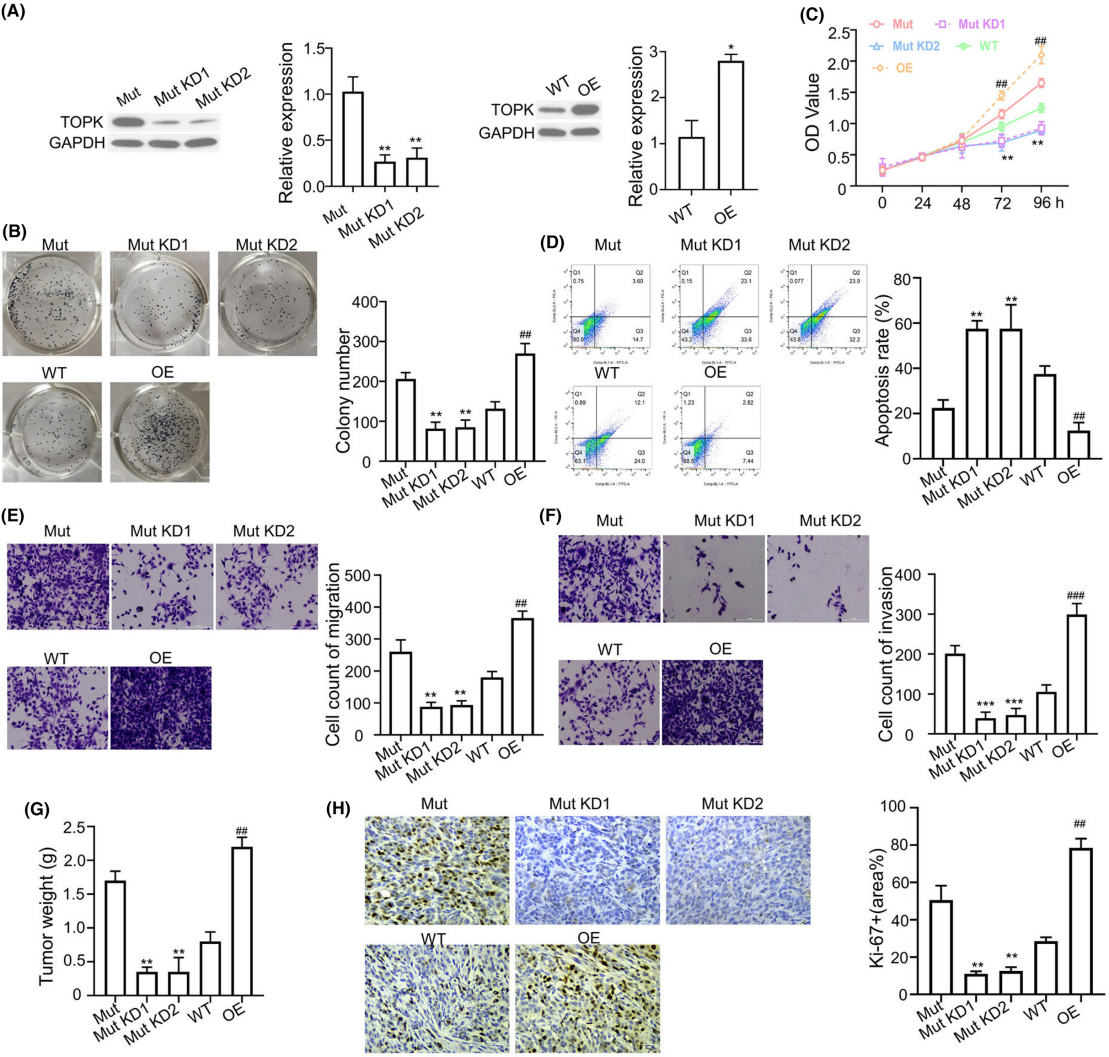
TOPK is indispensable for the malignant phenotype of NSCLC cells with *KRAS* ^*G12C*^ mutation. (A). The expression of TOPK in *KRAS* ^*G12C*^ mutant A549 cells was silenced by lentivirus carrying TOPK shRNA (KD #1 and #2) and the TOPK was overexpressed (OE) in *KRAS* ^WT^ cells. The knockdown and overexpresison efficiency was validated by Western blot. (B). Colony formation assay in TOPK‐overexpressing *KRAS* ^WT^ cells or TOPK‐silenced *KRAS* ^G12C^ cells. (C). CCK8 proliferation assay in TOPK‐overexpressing *KRAS* ^WT^ cells or TOPK‐silenced *KRAS* ^G12C^ cells. (D). Annexin V and PI staining of apoptotic cells in TOPK‐overexpressing *KRAS* ^WT^ cells or TOPK‐silenced *KRAS* ^G12C^ cells. (E) Transwell migration and (F) invasion assay in TOPK‐overexpressing *KRAS* ^WT^ cells or TOPK‐silenced *KRAS* ^G12C^ cells. (E). Tumour weights were determined in xenograft model injected with TOPK‐overexpressing *KRAS* ^WT^ cells or TOPK‐silenced *KRAS* ^G12C^ cells. (F). Ki67 levels were examined by IHC in xenograft tissues. In all figures, Mut indicates the A549 cells with KRAS ^G12C^ overexpression, WT indicates A549 cells with KRAS ^WT^ overexpression. KD refers to TOPK knockdown in KRAS ^G12C^ cells and OE refers to TOPK overexpression in KRAS ^WT^ cells. Results are presented as means ± SD. *p < 0.05; **p < 0.01; ***p < 0.001

To investigate the functional role of TOPK in NSCLC cells, we applied lentivirus to stably silence TOPK in *KRAS* ^G12C^ A549 cells or overexpress TOPK in *KRAS* ^WT^ A549 cells. Western blot analysis showed the successful knockdown (KD) in *KRAS* ^G12C^ mutation cells and the overexpression of TOPK (OE) in *KRAS* ^WT^ A549 cells (Figure 2A). Colony formation assay and CCK8 proliferation assay demonstrated that silencing TOPK in *KRAS* ^G12C^ cells significantly suppressed the proliferation, whereas the overexpression of TOPK promoted cell proliferation in *KRAS* ^WT^ A549 cells (Figure 2B,C). Moreover, Annexin V and PI staining showed that the percentage of apoptotic cells was increased in TOPK‐silenced *KRAS* ^G12C^ A549 cells, while the overexpression of TOPK suppressed apoptosis in *KRAS* ^WT^ A549 cells (Figure 2D). We also compared the migratory and invasive capacity of A549 cells with TOPK silencing or overexpression. The results indicate that silencing TOPK in *KRAS* ^G12C^ cells impaired cell migration and invasion the proliferation, whereas the overexpression of TOPK enhanced cell mobility in *KRAS* ^WT^ A549 cells (Figure 2E,F).

To further examine the role of TOPK in tumorigenesis, we established the xenograft tumour model of *KRAS* ^G12C^ A549 cells with TOPK silencing or *KRAS* ^WT^ A549 cells with TOPK overexpression. As shown in Figure 2G, the tumour weights were significantly reduced in TOPK silenced groups of *KRAS* ^G12C^ A549 cells, and TOPK overexpression enhanced tumorigeneis in *KRAS* ^WT^ A549 cells. In line with this finding, an decreased Ki67 expression was also found in TOPK‐silenced tumours tissues; however, the expression of Ki67 was increased in xenograft tissues from TOPK‐overexpressing *KRAS* ^WT^ A549 cells (Figure 2H). Taken together, the findings suggest that TOPK mediates the oncogenic effect of *KRAS* ^G12C^ mutation in NSCLC.

### 3.3 TOPK expression is dependent on the activity of MAPK/ERK signalling in NSCLC cells

**FIGURE 3:**
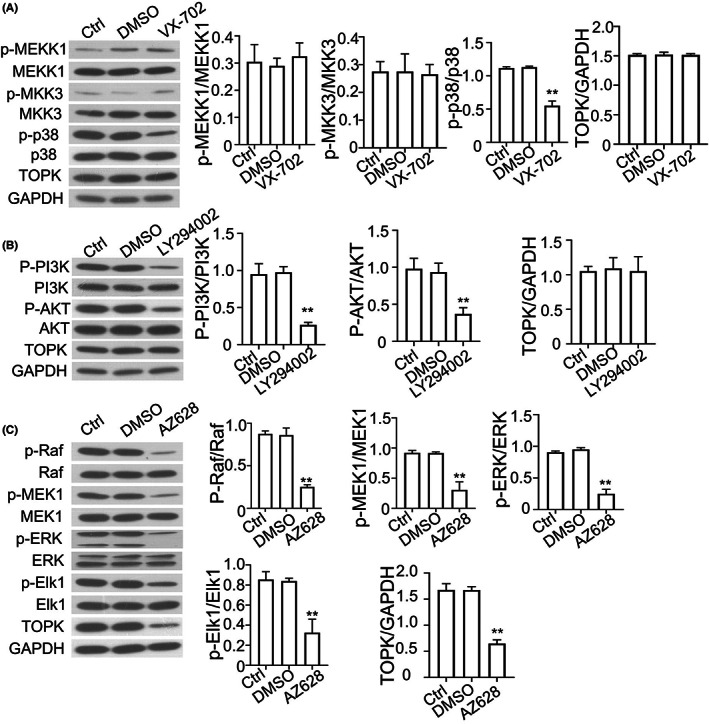
TOPK expression is regulated by MAPK/ERK signalling in NSCLC cells. (A). *KRAS* ^G12C^ A549 cells were treated with VX‐702 (p38 inhibitor), and the phosphorylation levels of MEKK1, MKK3, p38 and protein level of TOPK were detected by Western blot. The right panel showed the quantification of 3 experiments. (B). *KRAS* ^G12C^ A549 cells were treated with LY294002 (PI3K inhibitor), and the phosphorylation levels of PI3K, AKT and protein level of TOPK were detected by Western blot. The right panel showed the quantification of 3 experiments. (C). *KRAS* ^G12C^ A549 cells were treated with AZ628 (Raf inhibitor), the phosphorylation levels of Raf, MEK1, ERK and protein level of TOPK were detected by Western blot. The right panel showed the quantification of 3 experiments. Results are presented as means ± SD. *p < 0.05; **p < 0.01; ***p < 0.001

As KRAS is an upstream regulator of MAPK/ERK pathway, we hypothesized that TOPK expression is under the regulation of MAPK/ERK signalling pathway. We therefore treated *KRAS* ^G12C^ A549 cells with different inhibitors to determine which pathway is the major contributor to the TOK upregulation. As shown in Figure 3A, VX‐702 (p38 inhibitor) suppressed the phosphorylation of p38, but did not affect the phosphorylation level of MEKK1, MKK3 and had no effect on TOPK expression. LY294002, an inhibitor of PI3K, significantly decreased the phosphorylation level of PI3K and AKT, but did not reduce TOPK expression (Figure 3B). In addition, we detected the relationship between TOPK and MAPK/ERK signalling by using AZ628, which is widely used as a Raf inhibitor. The phosphorylation levels of Raf, MEK1, ERK and Elk1 were decreased upon AZ628 treatment, and TOPK expression level was also sigfnciantly decreased in AZ628 treated A549 cells (Figure 3C). Taken together, these findings suggest that the expression of TOPK is upregulated is dependent on the activity of MAPK/ERK signalling pathway in NSCLC cells.

### 3.4 TOPK is transcriptionally regulated by Elk1

**FIGURE 4:**
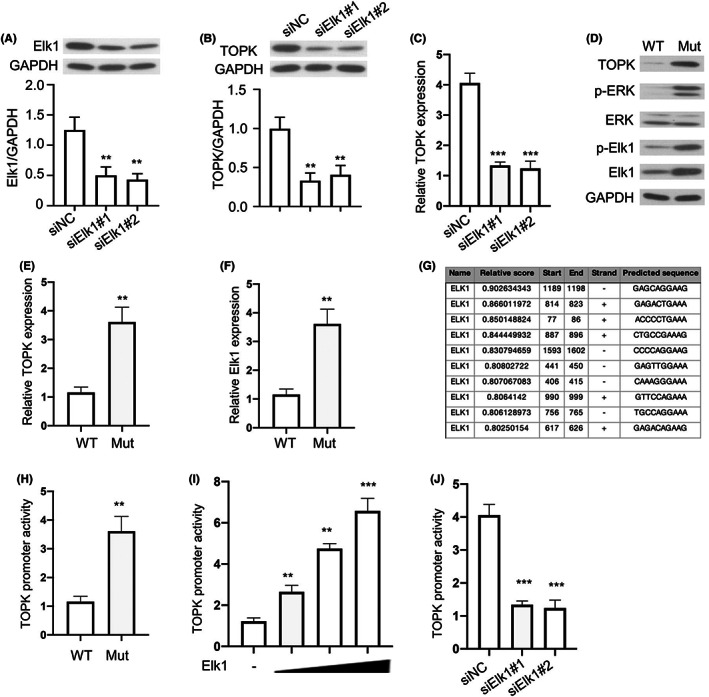
TOPK is transcriptionally regulated by Elk1. (A). *KRAS* ^G12C^ A549 cells was infected with lentivirus containing Elk1 shRNA and the knockdown efficiency was confirmed by Western blot. (B). TOPK protein level was detected in *KRAS* ^G12C^ A549 cells after Elk1 silencing. (C). TOPK mRNA level was detected in *KRAS* ^G12C^ A549 cells after Elk1 silencing. (D). Phosphorylation levels of ERK, Elk1 and protein level of TOPK in *KRAS* ^WT^ or *KRAS* ^G12C^ cells were detected by Western blot. (E). Quantification of TOPK expression in D. (F). Quantification of Elk1 expression in D. (G). Binding sites of Elk1 in TOPK promoter region predicted by JASPAR. (H). TOPK promoter activity was determined by dual luciferase activity assay in *KRAS* ^WT^ or *KRAS* ^G12C^ cells. (I). TOPK promoter activity was determined by dual luciferase activity assay in *KRAS* ^WT^ cells transfected with increasing dose of Elk1. (J). TOPK promoter activity in *KRAS* ^G12C^ cells upon Elk1 silencing. Mut indicates the A549 cells with KRAS ^G12C^ overexpression, WT indicates A549 cells with KRAS ^WT^ overexpression. Results are presented as means ± SD. *p < 0.05; **p < 0.01; ***p < 0.001

In Figure 3C, we observed that the phosphorylation level of Elk1 was suppressed and TOPK was downregulated by AZ628 treatment, and Elk1 was well‐known as a critical transcription factor downstream of MAPK/ERK signalling pathway.^24^ We assumed that TOPK could be transcriptionally regulated by Elk1. We therefore silenced Elk1 by lentivirus, and the knockdown efficiency was shown in Figure 4A. We found that the silencing of Elk1 decreased the expression of TOPK at both protein and mRNA levels (Figure 4B,C). In A549 cells with *KRAS* ^G12C^ mutation, the phosphorylation levels of Elk1 and ERK and the expression of TOPK were remarkably increased (Figure 4D,E). Furthermore, we also observed that the expression level of total Elk1 was enhanced by *KRAS* ^G12C^ mutation (Figure 4D,F), which was consistent with a previous report in colon cancer.^25^ In addition, ten putative Elk1‐binding sites were identified in the 2‐kb promoter of TOPK by an online tool JASPAR (http://jaspar.genereg.net) (Figure 4G). We therefore cloned the promoter region of TOPK gene locus into a luciferase reporter and performed the dual luciferase activity assay to examine the transcription activity of TOPK promoter. TOPK promoter activity in *KRAS* ^G12C^ was significantly higher in A549 cells with *KRAS* ^G12C^ mutation when compared to *KRAS* ^WT^ A549 cells (Figure 4H). Meanwhile, in *KRAS* ^WT^ A549 cells, TOPK promoter activity was increased by Elk1 in a dose‐dependent manner (Figure 4I), and the silencing of Elk1 dramatically suppressed the activity of TOPK promoter in *KRAS* ^G12C^ A549 cells (Figure 4J). Taken together, these findings suggest that TOPK was transcriptionally regulated by transcription factor Elk1.

### 3.5 TOPK promotes NF‐κB signalling activation via regulating TAK1 phosphorylation

**FIGURE 5:**
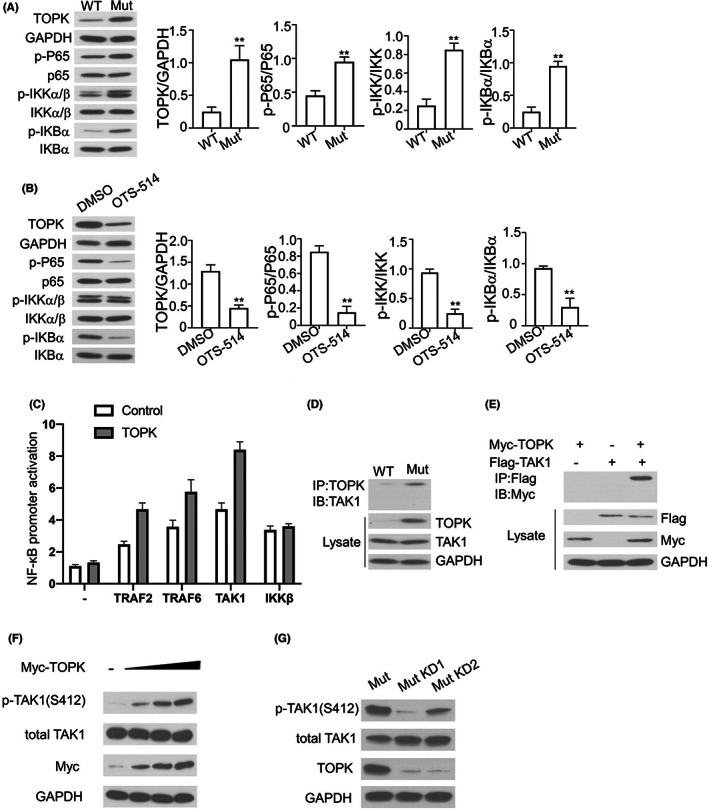
TOPK promotes NF‐κB signalling activation via regulating TAK1 phosphorylation. (A). Phosphorylation levels of p65, IKKβ, IκBα and protein level of TOPK in *KRAS* ^WT^ or *KRAS* ^G12C^ cells were detected by Western blot. The right panel showed the quantification of 3 experiments. (B). Phosphorylation levels of p65, IKKβ, IκBα and protein level of TOPK in *KRAS* ^G12C^ cells treated with DMSO or OTS514 (20 nM) were detected by Western blot. The right panel showed the quantification of 3 experiments. (C). NF‐κB promoter activity in *KRAS* ^WT^ A549 cells transfected with TRAF2, TRAF6 and TAK1 expression vector with/without TOPK overexpresison. (D). Interaction between TOPK and TAK1 in *KRAS* ^WT^ or *KRAS* ^G12C^ cells was detected by co‐IP analysis. (E). Myc‐TOPK and Flag‐TAK1 was expressed in *KRAS* ^WT^ cells, and their interaction was examined by anti‐flag IP experiment. (F). Phosphorylation level of TAK1 was detected by Western blot in *KRAS* ^WT^ cells with increasing dose of TOPK. (G). Phosphorylation level of TAK1 was detected by Western blot *KRAS* ^G12C^ cells upon TOPK silencing. Mut indicates the A549 cells with KRAS ^G12C^ overexpression, WT indicates A549 cells with KRAS ^WT^ overexpression. KD refers to TOPK silencing. Results are presented as means ± SD. *p < 0.05; **p < 0.01; ***p < 0.001

NF‐κB signalling pathway is one of the most crucial pathways implicated in the regulation of cell proliferation and tumorigenesis.^26^ Therefore we evaluated the activation status of NF‐κB signalling in *KRAS* ^WT^ and *KRAS* ^G12C^ A549 cells. As shown in Figure 5A, the phosphorylation levels of p65, IKKβ and IκBα were all increased in *KRAS* ^G12C^ cells. With the administration of OTS514 (a specific inhibitor for TOPK), the phosphorylation levels of p65, IKKβ and IκBα were significantly suppressed (Figure 5B). In addition, we examined the effect of TOPK on adaptor proteins (such as TRAF2, TRAF6, TAK1 and IKKβ)‐induced activation of NF‐κB transcription activity using a NF‐κB luciferase reporter. NF‐κB transcriptional activity was enhanced by TOPK overexpression in *KRAS* ^WT^ A549 cells transfected with TRAF2, TRAF6 and TAK1 expression vector, but TOPK overexpresison did not have effect in IKKβ expression cells, which indicated that TAK1 may be a potential target of TOPK (Figure 5C). Using the IP assay, we found that TOPK could physically interact with TAK1 in *KRAS* ^G12C^ cells (Figure 5D). Moreover, the interaction was confirmed by the overexpression of Myc‐TOPK and Flag‐TAK1 in *KRAS* ^WT^ cells (Figure 5E). Moreover, we found that that TOPK significantly enhanced the phosphorylation of TAK1 at S412 in a dose‐dependent manner (Figure 5F), and the silencing of TOPK decreased the level of phos‐TAK1 (Figure 5G). Taken together, these findings suggest that TOPK interacts with TAK1 and promotes TAK1 phosphorylation to activate NF‐κB signalling.

### 3.6 OTS514 enhances the anticancer effect of 5‐FU in *KRAS* ^*G12C*^ A549 cells

**FIGURE 6:**
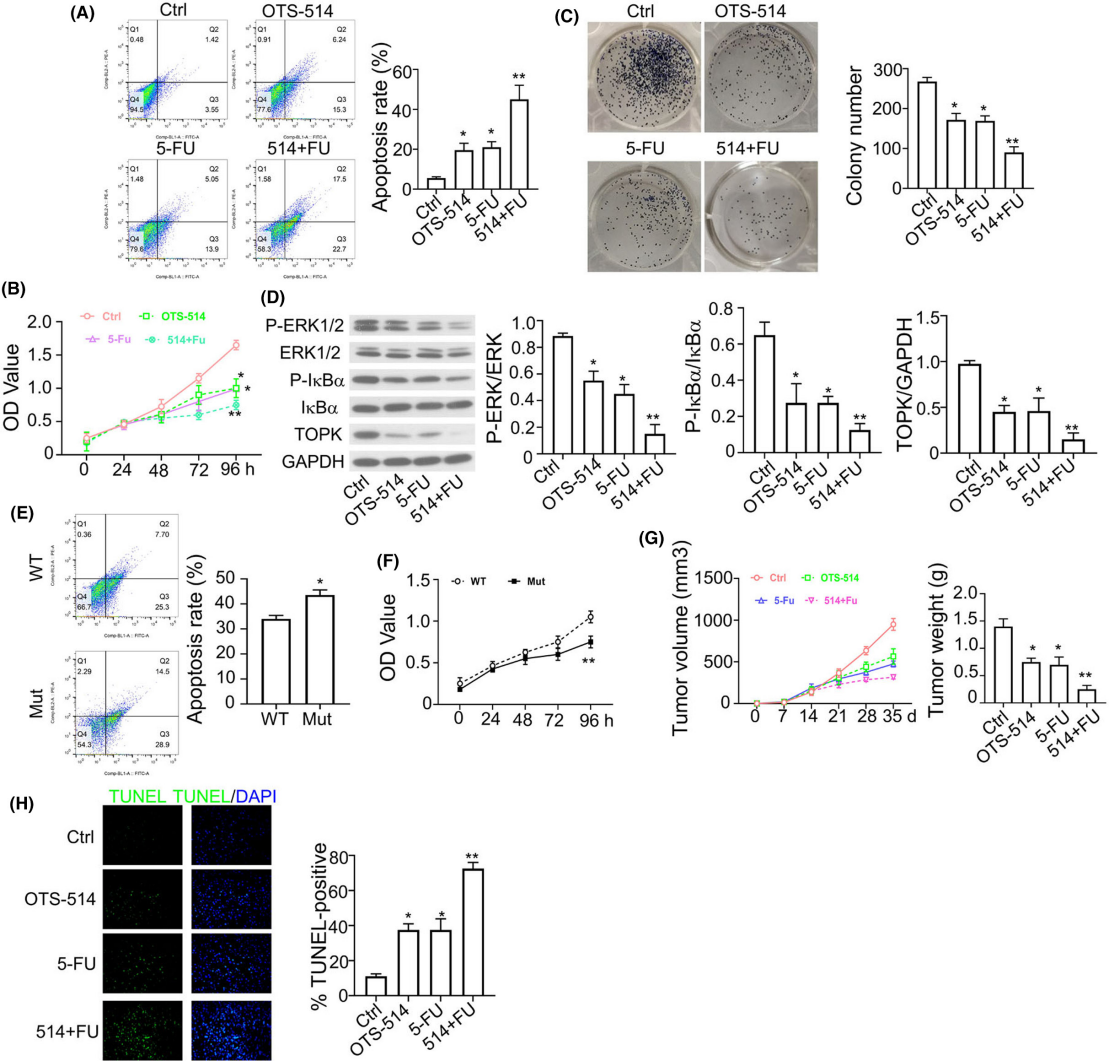
OTS514 enhances the anticancer effect of 5‐FU in *KRAS* ^G12C^ A549 cells. (A). Percentage of apoptotic events in *KRAS* ^G12C^ cells treated with OTS514 (20 nM) alone, 5‐FU (25 μM) alone or OTS514 + 5‐FU combination was examined flow cytometry. (B). CCK8 proliferation assay in *KRAS* ^G12C^ cells treated with OTS514 (20 nM) alone, 5‐FU (25 μM) alone or OTS514 + 5‐FU combination. (C). Colony formation assay in *KRAS* ^G12C^ cells treated with OTS514 (20 nM) alone, 5‐FU (25 μM) alone or OTS514 + 5‐FU combination. (D). Phosphorylation levels of ERK and IκBα in *KRAS* ^G12C^ cells treated with OTS514 (20 nM) alone, 5‐FU (25 μM) alone or OTS514 + 5‐FU combination were examined by Western blot. (E). Apoptotic events in *KRAS* ^WT^ or *KRAS* ^G12C^ cells treated with OTS514 + 5‐FU combination were measured by flow cytometry. Mut indicates the A549 cells with KRAS^G12C^ overexpression, WT indicates A549 cells with KRAS ^WT^ overexpression. (F). CCK8 proliferation assay in *KRAS* ^WT^ or *KRAS* ^G12C^ cells treated with OTS514 + 5‐FU combination. (G). Tumour volume and tumour weight in the xenograft model of *KRAS* ^G12C^ cells treated with OTS514 (2.5 mg/kg) alone or 5‐FU (100 mg/kg) alone or OTS514 + 5‐FU combination. (H). TUNEL staining of xenograft sections from mice treated with OTS514 (2.5 mg/kg) alone or 5‐FU (100 mg/kg) alone or OTS514 + 5‐FU combination. Results are presented as means ± SD. *p < 0.05; **p < 0.01; ***p < 0.001

5‐FU is a widely used chemotherapy agent in NSCLC treatment; however its application is constrained by the development of drug resistance.^27^, ^28^ To test whether TOPK regulates the sensitivity to 5‐FU induced anticancer effect, we treated *KRAS* ^G12C^ A549 cells with TOPK inhibitor OTS514, 5‐FU or in combination. As shown in Figure 6A, we found that OTS514 and 5‐FU could induce apoptosis in *KRAS* ^G12C^ cells, and the combination of OTS514 and 5‐FU showed strong synergistic effect in apoptosis induction. A similar result was observed in colony formation assay and CCK‐8 proliferation assay (Figure 6B,C). In addition, the phosphorylation levels of ERK and IκBα were strongly decreased in *KRAS* ^G12C^ cells treated with OTS514 + 5‐FU combination (Figure 6D). Furthermore, we found that the combination of OTS514 + 5‐FU induced more apoptotic cells and showed a more profound inhibition on cell proliferation in *KRAS* ^G12C^ cells when compared with *KRAS* ^WT^ cells (Figure 6E,F). The synergistic effect of OTS514 and 5‐FU was also validated in xenograft tumour model (Figure 6G), and the TUNEL staining in xenograft tumour section showed that the combination of OTS514 and 5‐FU administration showed strong synergistic effect in apoptosis induction (Figure 6H). Taken together, these findings suggest that TOPK inhibitor OTS514 could enhanced the effect of 5‐FU against NSCLC with *KRAS* ^G12C^ mutation.

### 3.7 Combination of OTS514 and AMG510 shows anti‐tumour effect in *KRAS* ^*G12C*^ A549 cells

**FIGURE 7:**
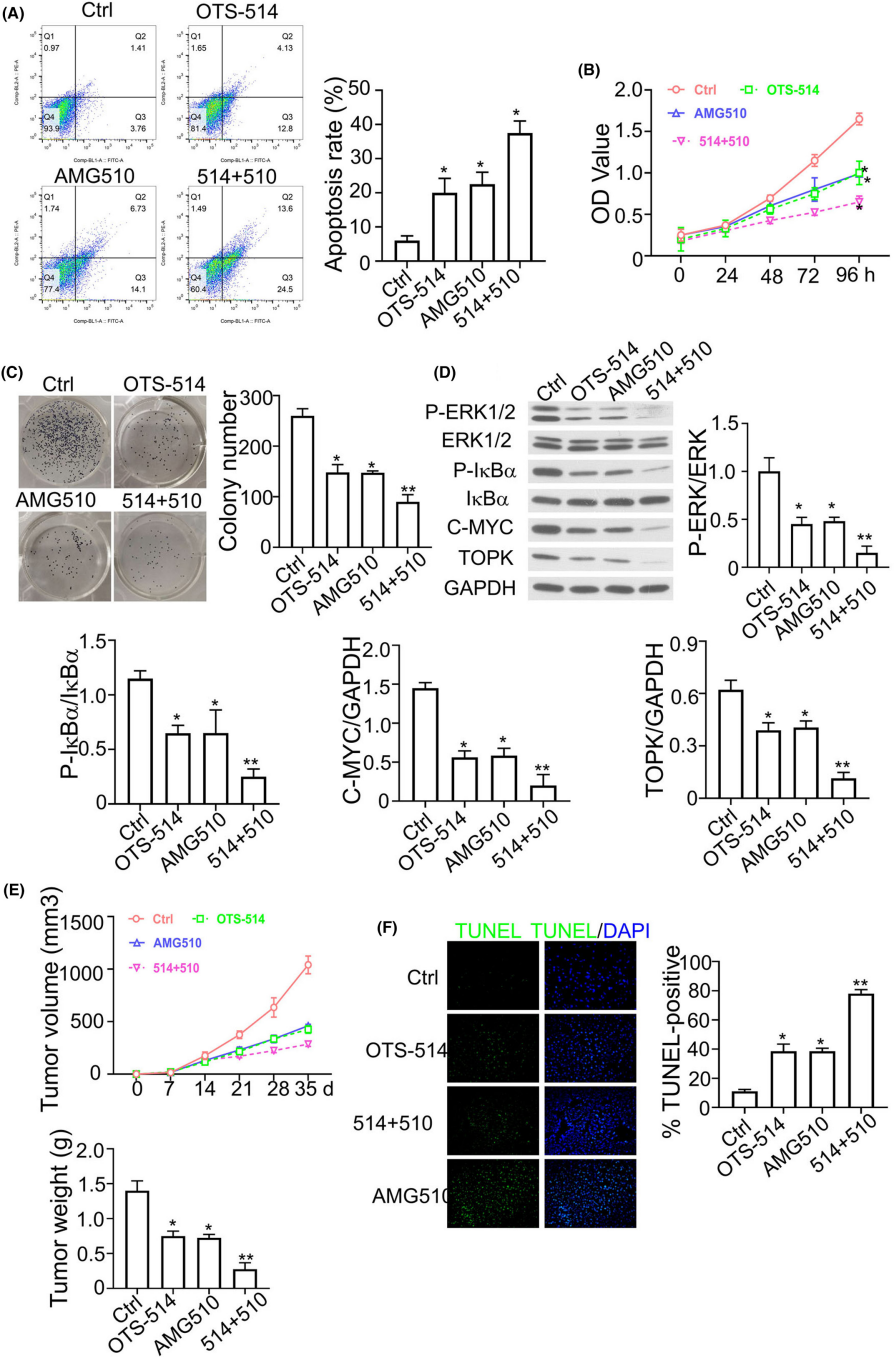
Combination of OTS514 and AMG510 shows synergistic anti‐tumour effect in *KRAS* ^G12C^ A549 cells. (A). Apoptotic events in *KRAS* ^G12C^ cells treated with OTS514 (20 nM) alone or AMG510 (1 μM) alone or OTS514 + AMG510 combination. (B). CCK8 proliferation assay in *KRAS* ^G12C^ cells treated with OTS514 (20 nM) alone or AMG510 (1 μM) alone or OTS514 + AMG510 combination. (C). Colony formation assay in *KRAS* ^G12C^ cells treated with OTS514 (20 nM) alone or AMG510 (1 μM) alone or OTS514 + AMG510 combination. (D). Phosphorylation levels of ERK and IκBα in *KRAS* ^G12C^ cells treated with OTS514 (20 nM) alone or AMG510 (1 μM) alone or OTS514 + AMG510 combination. (E). Tumour volume and tumour weight in the xenograft model of *KRAS* ^G12C^ cells treated with OTS514 (20 nM) alone or AMG510 (1 μM) alone or OTS514 + AMG510 combination. (F). TUNEL staining of xenograft sections from mice with OTS514 (20 nM) alone or AMG510 (1 μM) alone or OTS514 + AMG510 combination. Results are presented as means ± SD. *p < 0.05; **p < 0.01; ***p < 0.001

AMG510 is the first inhibitory agent that selectively targets *KRAS* ^G12C^ mutation.^8^ We further examined the anticancer effect of the combination of OTS514 and AMG510 on NSCLC. As shown in Figure 7A, the highest percentage of apoptosis was found in *KRAS* ^G12C^ cells treated with OTS514 + AMG510 combination. Cell proliferation was also significantly suppressed by the combination of OTS514 and AMG510 (Figure 7B,C). Consistently, the phosphorylation levels of ERK and IκBα were greatly decreased by the simultaneous treatment of OTS514 and AMG510 (Figure 7D). In the xenograft tumour model, OTS514 and AMG510 combination also showed strong synergistic effect against tumorigenesis, which was also evidenced by the increased apoptotic events in the tumour section (Figure 7E,F). Taken together, these findings suggest that the combinatory use of OTS514 and AMG510 could serve as an anticancer strategy against NSCLC cells with *KRAS* ^G12C^ mutation.

### 3.8 Evaluation of the anticancer effect of OTS514, AMG510 and 5‐FU combination in *KRAS* ^*G12C*^ A549 cells

**FIGURE 8:**
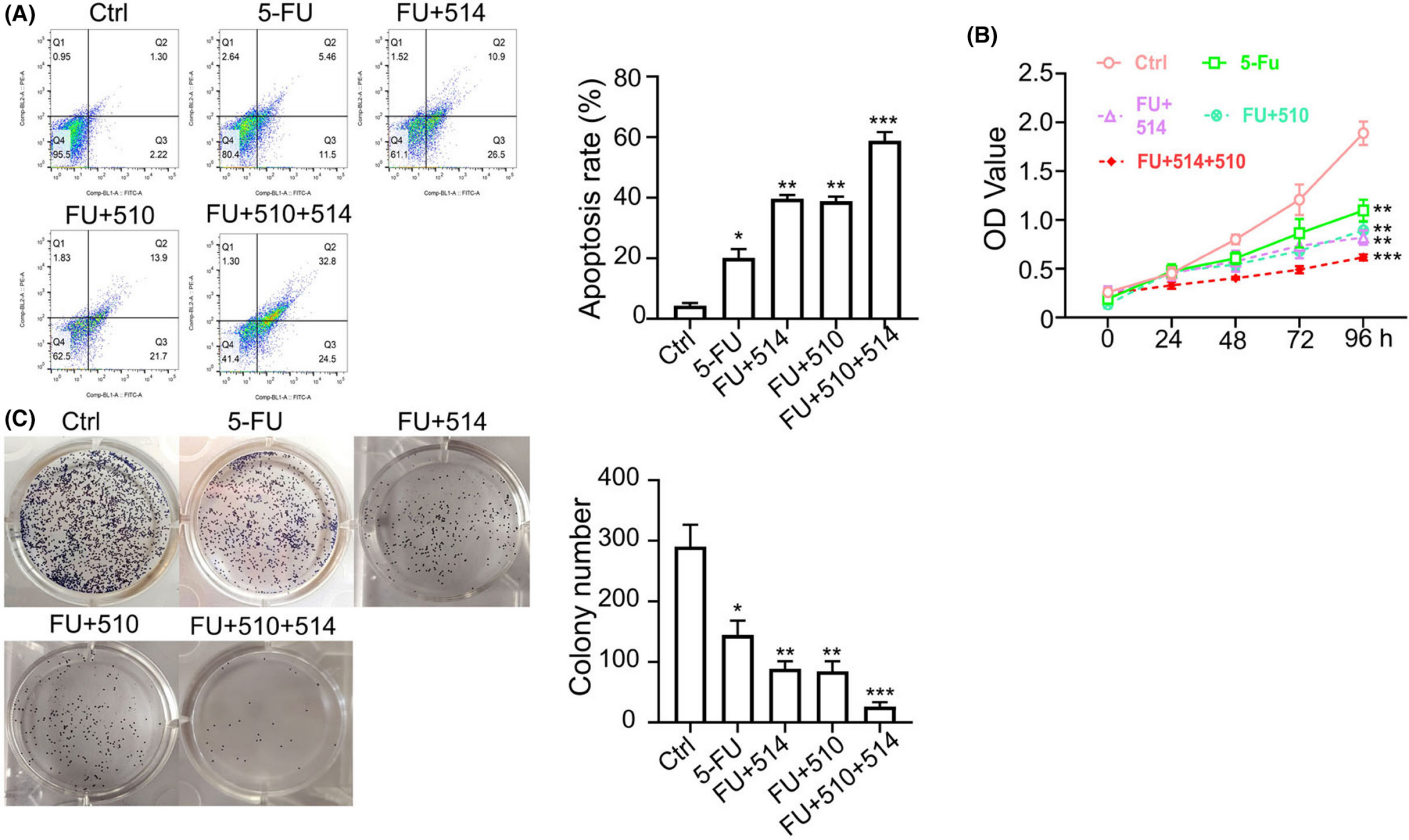
Evaluation of the anticancer effect of OTS514, AMG510 and 5‐FU combination in *KRAS* ^G12C^ A549 cells. (A) *KRAS* ^G12C^ cells treated with 5‐FU (25 μM) alone, 5‐FU + OTS514 (20 nM), or 5‐FU + AMG510 (1 μM) or 5‐FU + OTS514 + AMG510 combination. (A). Apoptotic events in *KRAS* ^G12C^ cells under above conditions were measured by flow cytometry. (B). CCK8 proliferation assay in *KRAS* ^G12C^ cells with the above treatments. (C). Colony formation assay in *KRAS* ^G12C^ cells in *KRAS* ^G12C^ cells with the above treatments. Results are presented as means ± SD. *p < 0.05; **p < 0.01; ***p < 0.001

To further evaluate the anticancer potential of the combination of OTS514, AMG510 and 5‐FU, we treated *KRAS* ^G12C^ A549 cells with different combination of drugs and examined the cell death and proliferation. As expected, we observed that the combinations of 5‐FU + OTS514 and 5‐FU + AMG510 both showed greater anticancer effect in *KRAS* ^G12C^ A549 cells in comparison to the 5‐FU treatment alone (Figure 8A–C). Moreover, when the three agents were applied together (5‐FU + OTS514 + AMG510), an even stronger apoptosis induction and anti‐proliferation effects were observed (Figure 8A–C). Therefore, the combinatory usage of 5‐FU, OTS514 and AMG510 could potentially produce the optimal anticancer effect against NSCLC with *KRAS* ^G12C^ mutation.

## 4 DISCUSSION

In this study, we investigated the relationship between *KRAS* ^G12C^ mutation and TOPK expression in NSCLC and found that, *KRAS* ^G12C^ mutation induced TOPK expression by activating MAPK/ERK signalling pathway and increasing the activity of transcription factor Elk1 in NSCLC. Importantly, we demonstrated the synergistic anticancer effect of TOPK1 inhibitor OTS514 and 5‐FU or the combination of OTS514 and AMG510 in NSCLC cells with *KRAS* ^G12C^ mutation, which provide insights into the formulation of novel therapeutic scheme for NSCLC treatment.

*KRAS* is one of the most common oncogenes in solid malignancies, and *KRAS* mutations exist in nearly 30% of tumours.^29^ Scientists have been making efforts in developing small molecule inhibitors for *KRAS* mutation for decades, but there are still no effective agents for the first‐line clinical treatment of tumours with *KRAS* mutations.^30^ Although AMG510 has been proved to be effective for the treatment of NSCLC with *KRAS* ^G12C^ mutation in clinical trials, its effect on other types of tumours with *KRAS* ^G12C^ mutation is limited. Therefore, understanding the molecular signalling downstream of *KRAS* ^G12C^ mutation is crucial for the design of novel combinatory drug treatment to enhance therapeutic effect of existing *KRAS* ^G12C^ inhibitors. Previous studies showed that *KRAS* ^G12C^ mutation induces the abnormal activation of various signalling pathways such as PI3K/Akt, MAPK/ERK and RALGDS/RAL^7^ and the hyper‐activation of transcription factors including Elk1 and NF‐κB,^25^, ^31^, ^32^ which are essential oncogenic signals for the tumorigenesis and tumour progression. Consistently, our results suggest that *KRAS* ^G12C^ mutation in NSCLC cells mediates the activation of MAPK/ERK signalling, thereby promoting the phosphorylation of transcription factor Elk1. The phosphorylated Elk1 shows enhanced transcriptional activity and induce a stronger transcriptional expression of TOPK. These data indicate that TOPK as a downstream effector of KRAS/MAPK/ERK/Elk1 axis.

T‐LAK cell‐originated protein kinase has been recognized as an oncogenic protein in various kinds of tumours.^33^ Mechanistically, TOPK was found to mediate hypoxia‐induced epithelial‐mesenchymal transition (EMT) and the invasion of NSCLC cells via the HIF‐1a/snail axis,^17^ and promote the EMT and invasion of breast cancer cells by upregulating TBX3 in TGF‐β1/Smad signalling.^10^ In addition, TOPK suppresses p53‐mediated transcription of pro‐apoptotic proteins to inhibit cell death in tumour cells.^34^ TOPK can also promote breast cancer cell proliferation by targeting geranylgeranylation signalling.^35^ However, the role of TOPK in NSCLC and the relationship between TOPK and *KRAS* ^G12C^ mutation remain largely unknown. In the current study, we reported that TOPK is significantly upregulated in NSCLC tissues and A549 cells with *KRAS* ^G12C^ mutation. Its overexpression is induced mainly by MAPK/ERK signalling and regulated by transcription factor Elk1. Importantly, TOPK overexpression not only supports the malignant phenotype of NSCLC cells in vitro, but also promotes the tumorigenesis of NSCLC cells in mouse model. Furthermore, we also observed that TOPK could activate NF‐κB signalling by interacting with TAK1 and phosphorylating TAK1. The enhanced NF‐κB signalling could also confer survival advantages in NSCLC cells.

Combination of different chemotherapeutics has been suggested to be effective approaches to enhance anticancer effect, and the proportion of combined drug usage in cancer treatment has increased in recent years.^36^, ^37^ In the current research, we found that the combination of OTS514 (TOPK inhibitor) and 5‐FU synergistically induces cell apoptosis and strongly suppresses the proliferation of *KRAS* ^G12C^ A549 cells. Moreover, compared to *KRAS* ^WT^ cells, *KRAS* ^G12C^ cells seem to be more sensitive for the combined treatment of OTS514 and 5‐FU. Furthermore, the combination of OTS514 and AMG510 (*KRAS* ^G12C^ mutant inhibitor) also shows strong synergistic effect against NSCLC cells with *KRAS* ^G12C^ mutation. When the three agents were applied together, the effect was further boosted with a profound effect in apoptosis induction and cell growth inhibition. These results together indicate that as a downstream of KRAS, TOPK is highly expressed in NSCLC cells with *KRAS* ^G12C^ mutation. The combinatory targeting of KRAS mutation and TOPK in NSCLC cells could be of great significance for overcoming the drug resistance of 5‐FU, which needs to be further evaluated in clinical practice.

**FIGURE 9:**
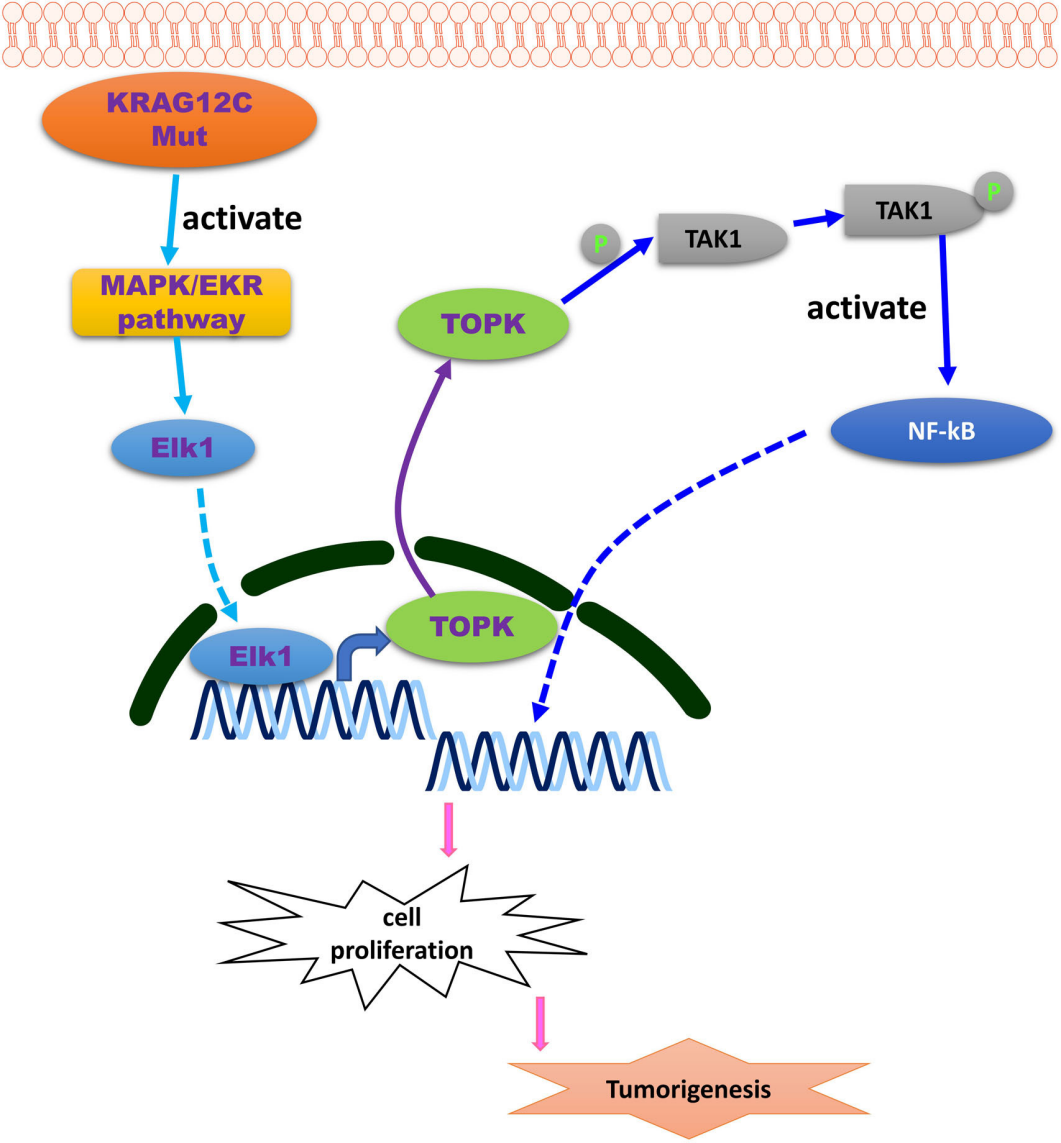
Schematic illustration of KRASG12C‐induced TOPK overexpression in tumour progression of NSCLC. TOPK induces the phosphorylation of TAK1 and leads to the continuous activation of NF‐κB signalling, resulting in the malignant progression of NSCLC cell

In conclusion, we reported the regulation of TOPK in NSCLC tissue and cell with *KRAS* ^G12C^ mutation and demonstrated that the overexpressed of TOPK is regulated by the hyper‐activation of MAPK/ERK/Elk1 axis (Figure 9). TOPK can induce the phosphorylation of TAK1 and leads to the continuous activation of NF‐κB signalling, resulting in the promotion of NSCLC cell proliferation. In the in vivo tumorigenesis model, the administration of TOPK inhibitor OTS514 enhanced the anticancer effect of 5‐FU, and the combinatory use of OTS514 and *KRAS* ^G12C^ inhibitor AMG510 showed synergistic anti‐tumour effect. Our results indicate that KRAS‐TOPK axis contributes to the progression of NSCLC and targeting this axis could synergize with anticancer effect of the existing chemotherapeutics for NSCLC treatment.

## AUTHOR CONTRIBUTIONS

**Chang Cai:** Methodology (lead); data curation (lead); formal analysis (lead); writing – original draft (lead); project administration (lead). **Shuo Yao:** Methodology (lead); writing – original draft (lead); writing – review and editing (lead). **Yanmei Zou:** Methodology (lead); writing – original draft (lead); writing – review and editing (lead). **Hui Lu:** Data curation (equal); formal analysis (equal); supervision (equal). **Xiuqiong Chen:** Data curation (equal); formal analysis (equal); investigation (equal). **Yali Wang:** Formal analysis (equal); validation (equal). **Kun Zheng:** Formal analysis (equal); visualization (equal). **Feng Zhu:** Investigation (equal); resources (equal). **Yihua Wang:** Conceptualization (lead); data curation (lead); project administration (equal); writing – review and editing (equal). **Hua Xiong:** Conceptualization (lead); data curation (lead); project administration (equal); writing – review and editing (equal). **Junfei Zhu:** Writing – review and editing (equal).

## FUNDING INFORMATION

None.

## CONFLICT OF INTEREST STATEMENT

The authors declare no competing financial interest.

## PATIENT CONSENT FOR PUBLICATION

Informed consent was obtained from all individual participants included in the study.

## Supporting information


FigureS1

TableS1

## Data Availability

The datasets used and/or analysed during the current study are available from the corresponding author on reasonable request.
