# Supplementary figures and images for: KRAS G12C mutation‐induced TOPK overexpression contributes to tumour progression in non‐small cell lung cancer
Source: J Cell Mol Med. 2023 May 24;27(12):1637–52. doi: 10.1111/jcmm.17640 (PMC10273069; doi:10.1111/jcmm.17640)

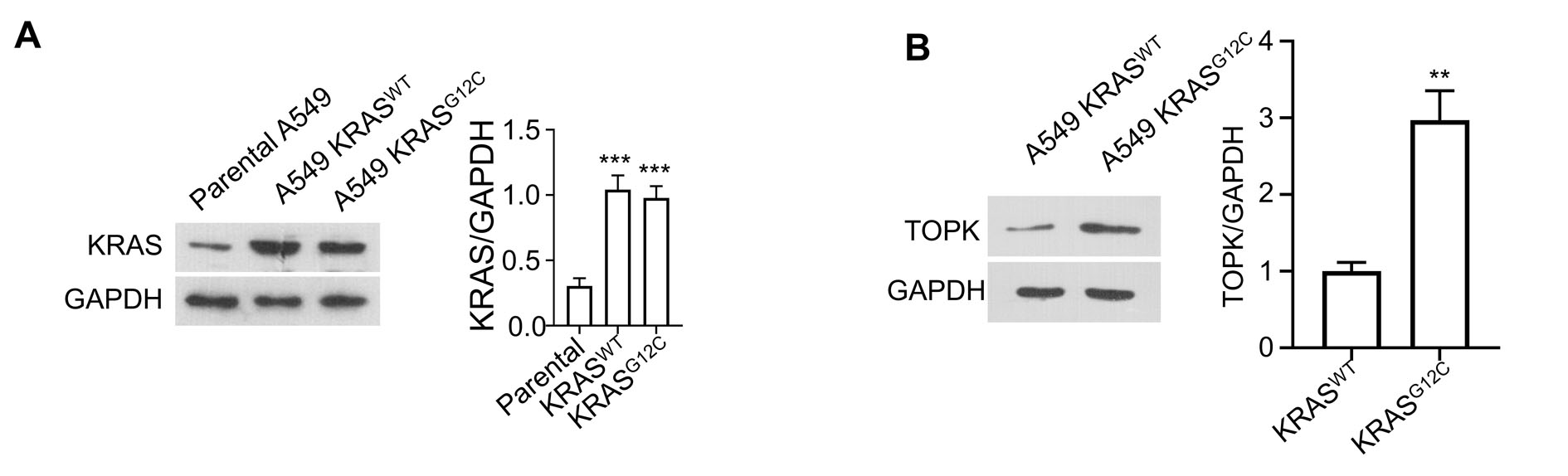

Supplement: Supplementary file 1 — FigureS1 [file JCMM-27-1637-s002.JPG]
